# Significance of circular RNAs in regulating protein ubiquitination for malignant tumor progression

**DOI:** 10.3389/fimmu.2025.1610112

**Published:** 2025-10-15

**Authors:** Haidong Zhong, Wei Li, Peiyue Luo, Qi Chen, Le Cheng, Lifeng Gan, Fangtao Zhang, Yiran Lu, Liying Zheng, Biao Qian

**Affiliations:** ^1^ The First Clinical College, Gannan Medical University, Ganzhou, Jiangxi, China; ^2^ Department of Urology, The First Affiliated Hospital of Gannan Medical University, Ganzhou, Jiangxi, China; ^3^ Key Laboratory of Urology and Andrology of Ganzhou, Ganzhou, Jiangxi, China; ^4^ Department of Graduate, The First Affiliated Hospital of Gannan Medical University, Ganzhou, Jiangxi, China

**Keywords:** malignant tumor, ubiquitination, circRNA, cancer therapy, immunotherapy

## Abstract

CircRNAs are an important class of non-coding RNAs, which are produced via back-splicing of exons and/or intron sequences of precursor mRNAs and generally cannot be translated into proteins as they do not bind to ribosomes. There is varying evidence supporting the claim that circRNAs are abnormally expressed in cancer and play a crucial role in cancer initiation and progression. Ubiquitin is a highly stable protein that can be conjugated to target proteins. The most crucial role of ubiquitination is to mediate the degradation of substrate proteins by the proteasome. An increasing amount of evidence indicates that circRNAs are involved in the precise degradation of proteins via the ubiquitin-proteasome system. This review systematically summarizes the intricate mechanisms by which circRNAs regulate target protein ubiquitination, modulate cancerous signaling pathways, and control tumorigenesis and tumor development. Although studies are continuously uncovering additional complex interactions between circRNAs and proteins, we believe that circRNAs are promising but challenging molecules that have the potential to facilitate precise cancer therapies in the future.

## 1 Introduction

CircRNAs are an important class of non-coding RNAs. They are generated through the back-splicing of exons and/or introns of precursor mRNAs. Additionally, they generally cannot be translated into proteins because they do not interact with ribosomes (1). Initially, circRNAs were considered to be abnormal products of spliceosome-mediated splicing errors (mis-splicing with scrambled exon orders),thus it is considered that circRNAs are unlikely to play a significant role in biological processes (2). However, now increasing evidence supports the diverse regulatory functions of circRNAs in various biological processes (3–5). As regulatory non-coding RNAs, circRNAs can directly participate in the regulation of gene expression and splicing events. Additionally, they can indirectly modulate other regulatory factors, such as competing with linear RNA splicing and miRNA sponge (3–5). Recent studies have revealed that circRNAs can exert their regulatory functions in malignant tumors, playing crucial roles in cancer initiation and progression. They can either promote cancer progression or act as tumor suppressors. For example, circRNAs can sustain proliferation signaling pathways to accelerate tumor growth, influence in signal pathways to regulate tumor metastasis (6), inhibit apoptosis to enhance chemotherapy drug resistance (7), and induce angiogenesis to facilitate tumor metastasis (8). Furthermore, circRNAs exhibit decent stability due to their covalent structure without polyA tails, which endows circRNAs with resistance to exonucleases (9). CircRNAs are abundant and stable in various tissues and display unique expression signatures associated with cancer progression and outcome. These characteristics render circRNAs potential diagnostic and prognostic biomarkers for cancer and potential therapeutic targets for malignant tumors (10, 11).

Ubiquitination is one of the most crucial post-translational modifications, which plays a significant role in maintaining the homeostasis of cellular proteins. Ubiquitin is widely expressed in eukaryotic cells and is a small protein composed of 76 residues (12). Ubiquitination of target proteins occurs as a result of a cascade of enzymatic reactions carried out by E1 (activating), E2 (conjugating), and E3 (ligating) enzymes. Finally, ubiquitin (Ub) is covalently coupled to lysine residues on target proteins (13). The ubiquitin-proteasome system (UPS) possesses a unique capacity to eliminate proteins in a highly specific manner and is implicated in nearly all aspects of cellular physiology and development (14). Ubiquitin-mediated proteolysis is essential for maintaining protein homeostasis (15). During cancer progression, proteins implicated in cancer progression can be specifically degraded by the ubiquitin-proteasome system, thereby influencing tumor growth and metastasis (16, 17). Consequently, the precise regulation of the ubiquitin-proteasome system is pivotal in the process of tumorigenesis.

Many studies have demonstrated that circRNAs participate in the development of malignant tumors through modulating the ubiquitin-proteasome system. Through these studies, we will gain a more comprehensive comprehension of the role that circRNAs play in regulating protein ubiquitination processes. In this review, we systematically elucidate the intricate mechanisms by which circRNAs modulate the ubiquitination of target proteins, influence signaling pathways and impact cancer development. Abundant evidence has demonstrated that circRNAs possess the potential to serve as biomarkers for cancer screening and prognosis. CircRNAs represent a class of molecules with great promise and challenges, which are expected to play a crucial role in the precise treatment of tumors in the future.

In recent years, an increasing number of studies have investigated the role of circular RNAs (circRNAs) in regulating ubiquitination and their implications in tumorigenesis; however, existing reviews either briefly discuss the role of circRNAs in regulating ubiquitination across multiple diseases or only provide a general overview of the circRNA-cancer relationship. To date, no systematic review has synthesized how circRNAs regulate protein ubiquitination and their subsequent impact on cancer progression across different cancer types. This review comprehensively synthesizes how circRNAs regulate ubiquitination, with a focus on their effects on malignant tumors (including cancers of the gastrointestinal tract, female reproductive system, and genitourinary system). The objectives of this review are to explore the pivotal role of circRNAs in regulating protein ubiquitination during malignant tumor progression and to highlight the potential value of circRNAs as diagnostic biomarkers and therapeutic intervention targets. By comprehensively evaluating and synthesizing the latest findings in this field, this review aims to advance the understanding of circRNA-mediated protein ubiquitination, thereby providing novel insights and strategies for the prevention, diagnosis, and treatment of malignant tumors.

## 2 Evidence acquisition and synthesis

We have systematically evaluated the latest research articles published in PubMed between 2015 and 2024, with PubMed serving as the primary database for literature retrieval. The following search strategies were used: ((“RNA, Circular”[Mesh]) AND (“Ubiquitination”[Mesh])), ((“RNA, Circular”[Mesh]) AND (“Neoplasms”[Mesh])), ((“Neoplasms”[Mesh]) AND (“Ubiquitination”[Mesh])), ((“RNA, Circular”[Mesh]) AND (“Neoplasms”[Mesh]) AND (“Ubiquitination”[Mesh])). We reviewed these manuscripts and prioritized studies that aligned with the review’s core focus (the regulatory relationship between circRNAs, ubiquitination, and malignant tumors) and those that were scientifically detailed and well-reported to help us understand them. Finally, 105 manuscripts were selected for inclusion in this review.

## 3 Gastrointestinal neoplasms

**Figure 1:**
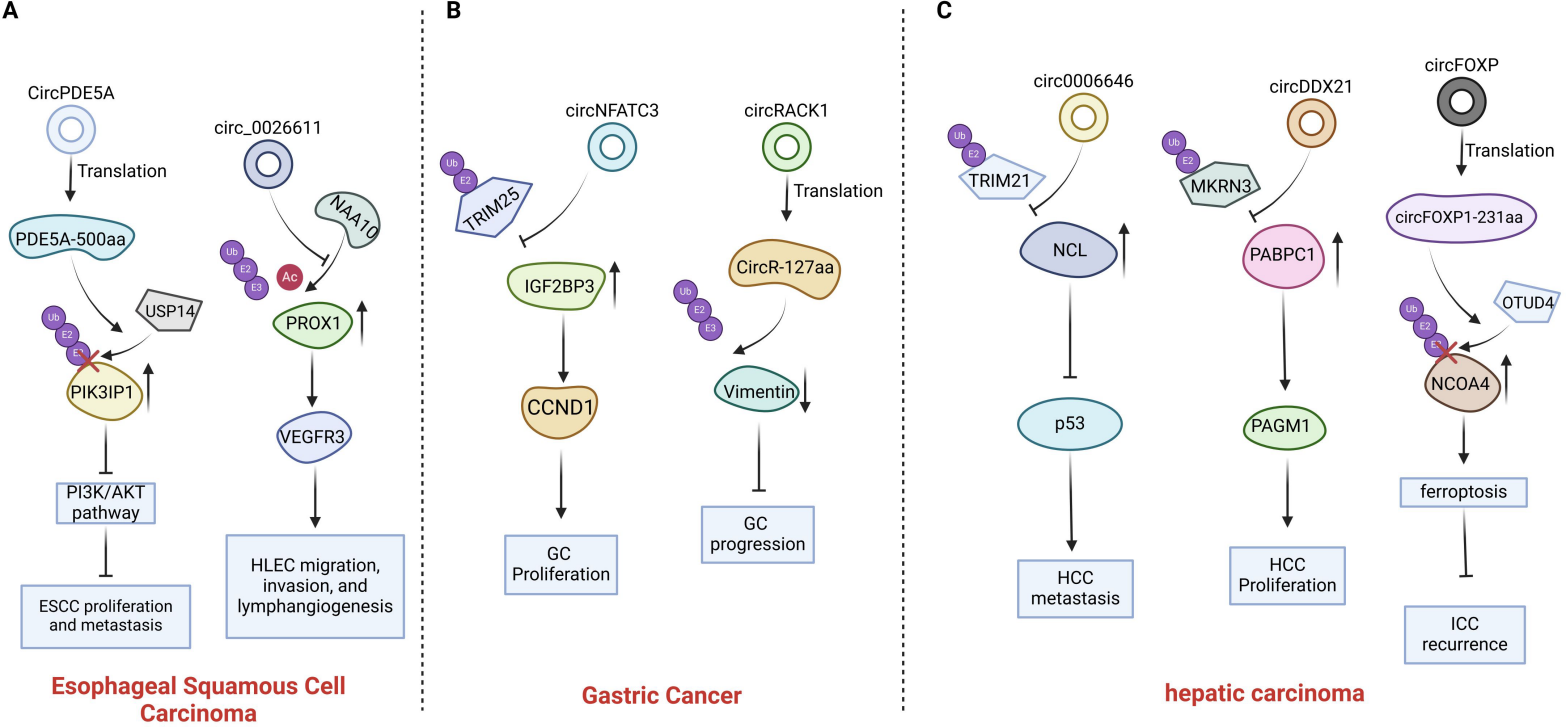
Introduction of circRNAs function on the esophagus cancer, gastric cancer and primary hepatic carcinoma. **(A)** CircPDE5A promotes USP14 mediated PIK3IP1 deubiquitination by encoding protein PDE5A-500aa; The interaction between circ0026611 and NAA10 impedes the binding of NAA10 to PROX1. **(B)** Circ NFATC3 binds directly to IGF2BP3, protecting IGF2BP3 from interacting with E3 ubiquitin ligase TRIM25; The protein circR-127aa, encoded by circRACK1, can regulate the ubiquitination of vimentin. **(C)** Circ0006646 obstructs the interaction between NCL and the E3 ubiquitin ligase TRIM21; CircDDX21 inhibiting MKRN3-mediated ubiquitination of PABPC1; circFOXP1 promotes OTUD4-mediated deubiquitination of NCOA4 by encoding protein circFOXP1-231aa.

**Figure 2:**
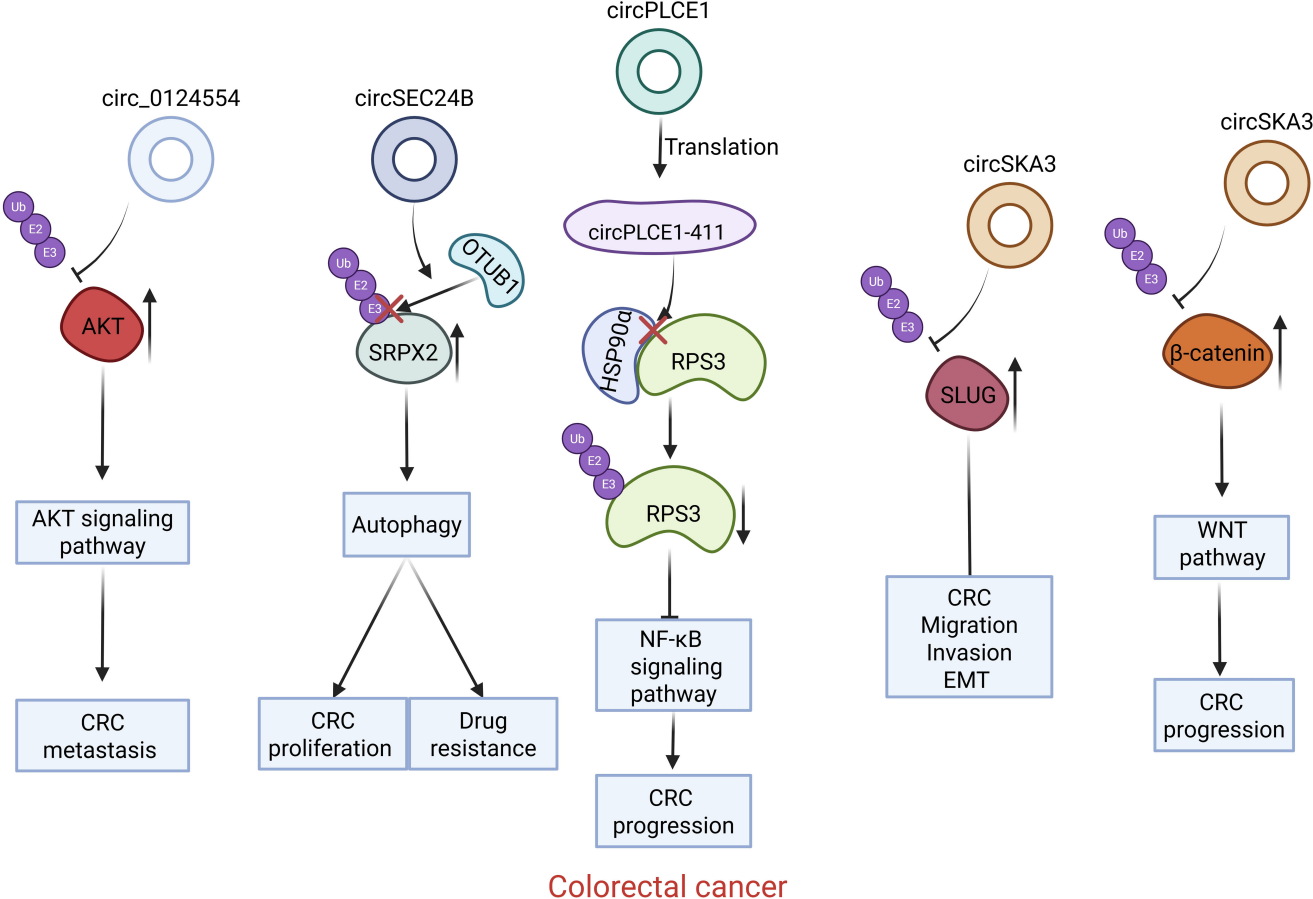
Introduction of circRNAs function on the colorectal carcinoma. Circ0124554 directly interacts with AKT to regulate the ubiquitination-mediated degradation of AKT; circSEC24B facilitates the interaction between SRPX2 and OTUB1 promotes the deubiquitination of SRPX2; The circPLCE1 encoded protein circPLCE1–411 binds to HSP90α, prompting RPS3 dissociation from the HSP90α/RPS3 complex, removing its protection, and triggering ubiquitin-dependent degradation of RPS3; circSKA3 interacts with SLUG and prevents its ubiquitination; CircSKA3 directly binds to β-catenin and inhibits its ubiquitination.

Gastrointestinal cancers include esophageal cancer (EC), gastric cancer (GC), hepatocellular carcinoma (HCC), cholangiocarcinoma (CCA), pancreatic cancer (PC) and colorectal cancer (CRC). Recent reports indicate that circRNAs exhibit abnormal expression in gastrointestinal neoplasms. CircRNAs with abnormal expression influence gastrointestinal cancer through modulating multiple mechanisms (18). Multiple circRNAs have been identified to affect gastrointestinal cancers development through regulating ubiquitination of crucial oncoproteins or tumor suppressive proteins (**Figures 1**, **2**, **Table 1**).

**Table 1:** Effects of circRNA on gastrointestinal cancers.

Disease	CircRNA	Protein ubiquitination level	Signaling pathways	Function	Ref
Esophagus cancer	CircPDE5A	Reduce the ubiquitination level of PIK3IP1	PI3K/AKT	Inhibit tumor proliferation and metastasis	(19)
	Circ0026611	Reduce the ubiquitination level of PROX1	lymphatic transcription factors	Enhance the migration, invasion and lymphangiogenesis of human lymphatic endothelial cells	(20)
Gastric cancer	CircNFATC3	Reduce the ubiquitination level of IGF2BP3	Non-coding RNA	Promoting tumor progression	(21)
	CircRACK1	Increase the ubiquitination level of vimentin	EMT	Inhibition of gastric cancer cell metastasis	(22)
Colorectal carcinoma	Circ0124554	Reduce the ubiquitination level of AKT	AKT	Promote tumor cell proliferation and inhibit apoptosis	(23)
	CircSEC24B	Reduce the ubiquitination level of SRPX2	FAK/SRC/ERK	Promote the proliferation of CRC cells and activate autophagy to induce chemotherapy resistance	(24)
	CircPLCE1	Increase the ubiquitination level of RPS3	NF-κB	Inhibit the proliferation and metastasis of CRC cells	(25)
	CircSKA3	Reduce the ubiquitination level of SLUG	EMT	Promote EMT, metastasis and invasion of CRC cells	(26)
	CircSKA3	Reduce the ubiquitination level of β-catenin	Wnt/β-catenin	Promote the proliferation and migration of CRC cells	(27)
Primary Hepatic Carcinoma	Circ0006646	Reduce the ubiquitination level of NCL	p53	Enhance the metastasis of HCC	(28)
	CircDDX21	Reduce the ubiquitination level of PABPC1	glycolysis	Promoting the growth of hepatocellular carcinoma cells *in vivo*	(29)
	CircFOXP	Reduce the ubiquitination level of NCOA4	Apoptosis	Inhibit the proliferation, colony formation and invasion of ICC cells	(30)
	CircCDYL	Reduce the ubiquitination level of HRNR	mTOR-p70S6K	Reducing the sensitivity of HCC cells to anti-PD-L1 therapy and sustaining tumor invasiveness	(31)
	CircCCNY	Increase the ubiquitination level of HSP60	MAPK and HSP60/c-myc/PD-L1	Inhibiting tumor immune evasion in HCC and enhancing sensitivity to lenvatinib treatment.	(32)

### 3.1 Esophageal cancer

#### 3.1.1 CircPDE5A

Through the screening of differentially expressed circular RNAs (circRNAs) in human esophageal squamous cell carcinoma (ESCC) tissues, Lei et al. discovered that CircPDE5A was significantly down-regulated in the cancer tissues of ESCC patients when compared to the adjacent normal tissues. Moreover, patients with low expression levels of CircPDE5A exhibited a more advanced clinicopathological stage and a poorer prognosis. Consistently, both *in vitro* and *in vivo* experiments demonstrated that the silencing of circPDE5A significantly enhanced the proliferation and metastatic potential of ESCC cells (19). In contrast to other non-coding circRNAs, CircPDE5A encodes a newly discovered protein PDE5A-500aa that inhibits the proliferation and metastasis ability of esophageal cancer cells both invitro and *in vivo* (19). The specific mechanism is that the protein PDE5A-500aa has the ability to stabilize the expression level of phosphatidylinositol-3-kinase interacting protein 1 (PIK3IP1) by facilitating the USP14-mediated deubiquitination process of PIK3IP1; Ubiquitin-specific peptidase 14 (USP14) is a deubiquitinating enzyme that catalyzes the removal of ubiquitin moieties from target proteins (33). PIK3IP1 binds to PI3K and prevents its activation, thereby inhibiting PI3K activity, ultimately resulting in the suppression of tumor cell proliferation and metastasis (34). In summary, circPDE5A promotes the USP14-mediated deubiquitination of PIK3IP1 through encoding the protein PDE5A-500aa. This process consequently attenuates the PI3K/AKT signaling pathway in ESCC cells, leading to the inhibition of ESCC cell proliferation and metastasis (19).

#### 3.1.2 Circ0026611

In comparison with normal esophageal epithelial cells, circular RNA 0026611 (circ0026611) was upregulated in esophageal squamous cell carcinoma (ESCC) cells and exosomes derived from those ESCC cells. Furthermore, exosomes derived from ESCC cells transmitted circ0026611 and thereby promoted lymphangiogenesis *in vitro*, and the overexpression of circ0026611 could enhance the migratory, invasive, and tube-forming abilities of human lymphatic endothelial cells (HLECs). However, the existing of literature is insufficient to comprehensively validate its influence in ESCC. Prospero homeobox protein 1(PROX1) influences lymphangiogenesis by modulating the expression levels of diverse lymphatic transcription factors. For instance, it facilitates the upregulation of Vascular endothelial growth factor receptor 3(VEGFR3) (35). N-alpha-acetyltransferase 10(NAA10) is an acetyltransferase enzyme that catalyzes the acetylation of specific amino acid residues within proteins (36). NAA10 is capable of interacting with PROX1, thereby facilitating the acetylation of the PROX1 protein. Following this acetylation, the acetylation event promotes the ubiquitination of the PROX1 protein, which subsequently leads to its degradation by the proteasome system (20). The experimental results demonstrated that NAA10 facilitated the acetylation and subsequent ubiquitination of PROX1. The specific mechanism by which circ0026611 exerts its effect is that circ0026611 interacts with NAA10 without altering the expression level of NAA10. Subsequently, this interaction between circ0026611 and NAA10 inhibits the interaction between NAA10 and PROX1, leading to a decrease in the ubiquitination and acetylation of PROX1. Circ0026611 does not influence the synthesis of PROX1. However, it is capable of inhibiting the ubiquitination and acetylation processes of PROX1. By doing so, it reduces the degradation of PROX1 by the proteasome system. Consequently, circ0026611 exerts a positive regulatory effect on the PROX1 protein. In summary, circ0026611 stabilizes the expression of PROX1 by inhibiting the ubiquitination and acetylation of the PROX1 protein. This inhibition indirectly leads to the promotion of VEGFR3 expression. As a consequence, it enhances the migratory, invasive, and tube-forming abilities of HLECs (20).

### 3.2 Gastric cancer

#### 3.2.1 CircNFATC3

Yang et al. initially identified circRNAs that bind to IGF2BP3 in gastric cancer (GC) cells. Subsequently, they screened these circRNAs in 16 pairs of GC tissues and adjacent non-tumor tissues obtained from GC patients. They discovered that circNFATC3 was significantly upregulated in GC tissues and bound to IGF2BP3 when compared with adjacent normal tissues. The expression level of circNFATC3 in GC tissues exhibited a positive correlation with the volume of the tumor. Consistently, the silencing of circNFATC3 was found to inhibit the proliferation of GC cells both *in vitro* and *in vivo*. Insulin-like growth factor 2 mRNA-binding protein 3(IGF2BP3) is an RNA-binding protein that promotes tumor progression through its binding to non-coding RNAs (37, 38). Tripartite motif-containing protein 25 (TRIM25), an E3 ubiquitin ligase, mediates the ubiquitination of IGF2BP3 by interacting with the IGF2BP3 protein and subsequently degrades it via the ubiquitin-proteasome pathway (39). Moreover, circNFATC3 can interact with IGF2BP3 and block TRIM25-mediated ubiquitination, thereby inhibiting the degradation of IGF2BP3. The above results indicate that circNFATC3 inhibits the degradation of IGF2BP3 by interfering with the ubiquitin-proteasome pathway. The interaction between circNFATC3 and IGF2BP3 impedes the ubiquitination-mediated degradation of IGF2BP3. This is the primary mechanism by which circNFATC3 promotes the growth and proliferation of GC cells. In addition, circNFATC3 is able to bind to IGF2BP3, regulate the stability of cyclin D1 (CCND1) messenger RNA (mRNA) to enhance CCND1 expression level and subsequently promote the proliferation of GC cells through CCND1. In conclusion, circNFATC3 directly engages in binding with IGF2BP3. This binding action safeguards IGF2BP3 from interacting with the E3 ubiquitin ligase, TRIM25. As a consequence, it suppresses the TRIM25-dependent ubiquitination of IGF2BP3 and subsequently prevents the degradation of IGF2BP3 (21).

#### 3.2.2 CircRACK1

In gastric cancer (GC) cells, circRACK1 is one of the most prominently down-regulated circular RNAs (circRNAs). Qiao et al. discovered that the silencing of circRACK1 can promote the growth and metastasis of gastric cancer cells both *in vitro* and *in vivo*. These findings indicate that circRACK1 has an anti-tumor effect. Unlike other non-coding circRNAs, circRACK1 can encode a protein named CircR-127aa (22). The protein encoded by circular RNA associated with receptor for activated C kinase 1, CircR-127aa, can interact with vimentin and actin. These two proteins are both crucial components of the actin-cytoskeleton. Moreover, the remodeling of the actin-cytoskeleton plays a pivotal role in the motility and migration of tumor cells during the process of EMT (40). The interaction between the protein CircR-127aa encoded by circRACK1 and vimentin is capable of enhancing the ubiquitination of vimentin. This enhanced ubiquitination promotes the ubiquitin-mediated degradation of vimentin via the proteasome pathway, ultimately leading to the inhibition of vimentin expression. In conclusion, the protein CircR-127aa encoded by circRACK1 is able to regulate the ubiquitination of vimentin, which plays a role in modulating the remodeling of the actin-cytoskeleton, controlling the process of EMT, and ultimately exerts an anti-tumor effect (22). But no information regarding the E3 ligase of vimentin has been reported in this study.

### 3.3 Colorectal carcinoma

#### 3.3.1 Circ0124554

In colorectal cancer (CRC), metastasis is a widely recognized factor contributing to a poor prognosis. Tang et al. conducted a comparison of the differentially expressed circular RNAs (circRNAs) in the tumor tissues, adjacent tumor tissues, and normal tissues of colorectal cancer patients with synchronous liver metastasis (LNLM1) and those without liver metastasis (LNLM0). Their findings demonstrated that, when compared with colorectal cancer patients without liver metastasis, higher expression level of circular RNA Circ0124554 was detected in the tissues of colorectal cancer patients with liver metastasis. Additionally, the expression level of Circ0124554 was markedly higher in the tumor tissues of patients with liver metastasis (LNLM1) than in the adjacent tumor tissues and normal tissues. When compared with CRC patients exhibiting normal expression levels of Circ0124554, CRC patients with elevated expression levels of Circ0124554 had a poorer prognosis, irrespective of the presence or absence of liver metastasis. Additionally, the increased expression of Circ0124554 was correlated with vascular invasion and liver metastasis. Consistently, the silencing of Circ0124554 was found to significantly suppress the invasive ability of CRC cells. AKT is a serine-threonine kinase that plays a crucial role in promoting the proliferation of tumor cells and suppressing apoptosis (41). Circ0124554 can directly bind to the ubiquitination site of AKT. By that, it reduces the ubiquitination level of AKT, sustains the expression level of AKT, and consequently leads to the continuous activation of the AKT signaling pathway. With the overexpression of Circ0124554, the phosphorylation level of AKT, the expression levels of its downstream proteins, and the total expression level of AKT were all elevated (23). Consequently, within CRC cells, Circ0124554 directly interacts with AKT. This interaction regulates the ubiquitination-mediated degradation of AKT, leading to the continuous activation of AKT-associated signaling pathways and ultimately facilitating the metastasis of colorectal cancer.

#### 3.3.2 CircSEC24B

The activation of autophagy is of pivotal importance in modulating chemoresistance within colorectal cancer cells (42). Wang et al. conducted a screening for differentially expressed circular RNAs (circRNAs) in colorectal cancer (CRC) cells that were treated with autophagy agonists and in oxaliplatin-resistant CRC cells. They discovered that the expression of circSEC24B in CRC cells and tissues was continuously elevated compared to that in the corresponding normal specimens. Simultaneously, the expression level of circular RNA circSEC24B in oxaliplatin (OXA)-resistant CRC cells was notably elevated when compared to that in normal CRC cells. These findings suggest that circular RNA circSEC24B might play a role in autophagy activation and contribute to chemotherapy resistance in CRC cells (24). Furthermore, circular RNA circSEC24B is capable of directly interacting with OTUB1, and it can directly bind to Sushi repeat-containing protein(SRPX2). OTUB1 functions as a deubiquitinase. This protein, OTUB1, is capable of interacting with protein SRPX2, thereby leading to a reduction in the ubiquitination level of SRPX2. Circular RNA circSEC24B can directly bind to both OTUB1 and protein, SRPX2 simultaneously. Acting as a scaffold molecule between SRPX2 and OTUB1, circular RNA circSEC24B facilitates the interaction between these two proteins. This interaction promotes the deubiquitination of SRPX2 and ultimately reduces the degradation of SRPX2. Subsequently, SRPX2 will promote the progression of cancer through activating the FAK/SRC/ERK pathway (43, 44). In summary, circular RNA circSEC24B facilitates the proliferation of CRC cells and triggers the activation of autophagy, thereby inducing chemoresistance. It accomplishes this by promoting the deubiquitination of SRPX2 protein and stabilizing the SRPX2 protein (24).

#### 3.3.3 CircPLCE1

Through a comparison of the differentially expressed circular RNAs (circRNAs) in colorectal cancer (CRC) tissues and the corresponding normal tissues, Liang et al. found that the expression level of circular RNA circPLCE1 was reduced in CRC samples when contrasted with the corresponding normal samples. Moreover, a low expression level of circular RNA circPLCE1 was correlated with a poor survival rate for CRC patients. In contrast to other non-coding circRNAs, circular RNA circPLCE1 has the ability to encode a novel protein named circPLCE1-411. Moreover, the expression level of circPLCE1–411 shows a negative correlation with both the clinical stage of the tumor and the T stage (25). Consistently, the expression of circular RNA circPLCE1 suppresses the proliferation and metastasis of CRC cells *in vivo*. However, circular RNA circPLCE1 with an initial codon mutation exerts no impact on the proliferation of CRC cells. In contrast, the normal form of circular RNA circPLCE1 is capable of suppressing the migration and invasion of CRC cells. This observation suggests that circular RNA circPLCE1 inhibits the proliferation and migration of CRC cells through the regulation mediated by the encoding of the circPLCE1–411 protein, rather than through other regulatory mechanisms. RPS3 serves as a crucial regulator within the NF-κB signaling pathway. 40S ribosomal protein S3(RPS3) has the ability to augment the NF-κB signaling pathway and is involved in the physiological processes related to chemotherapy resistance, the proliferation of tumor cells, and the metastasis of diverse tumors (45–47); Heat shock protein 90 (HSP90) is capable of interacting with RPS3. Through the prevention of the ubiquitination of RPS3 and the proteasome-dependent degradation of RPS3, HSP90 enhances the stability of RPS3 (48). Heat shock protein 90 alpha (HSP90α) functions as a chaperone protein for RPS3. The circular RNA encoded protein circPLCE1–411 binds to HSP90α, facilitating the dissociation of RPS3 from the HSP90α/RPS3 complex. This dissociation leads to the removal of the protective effect on RPS3, triggering ubiquitin-dependent degradation of RPS3. Consequently, the NF-κB signaling pathway is inhibited. Consequently, circular RNA circPLCE1 suppresses the NF-κB signaling pathway. It does so by encoding the protein circPLCE1-411, which in turn reduces the expression level of RPS3 within CRC cells, thereby exerting its tumor-suppressive effect (25).

#### 3.3.4 CircSKA3

EMT, a pivotal biological process, is of great significance in the metastasis and dissemination of cancer cells (49). Zinc finger protein SNAI2 (SLUG) is a member of the Snail family of transcription factors. It serves as a crucial regulator of epithelial-mesenchymal transition (EMT) and plays an important role in initiating EMT (50, 51). Circular RNA circ0000467 is generated through the splicing and cyclization processes of exon 4 of the host gene SKA3, which is situated on human chromosome 13, so it is named circSKA3. When compared to the corresponding normal samples, the expression of circular RNA circSKA3 was upregulated in tumor samples. Moreover, elevated expression levels of circular RNA circSKA3 were significantly correlated with a poor prognosis for patients with CRC. Consistently, the silencing of circular RNA circSKA3 significantly inhibited the migration, invasion and metastasis capabilities of CRC cells and improved prognosis *in vitro* and *in vivo* (26). The transcription factor SLUG exhibits a short half-life and its degradation is precisely regulated by the ubiquitin-proteasome system. Circular RNA circSKA3 interacts with the transcription factor SLUG and, by impeding the ubiquitination process, maintains the stability of SLUG protein levels. In conclusion, circSKA3 facilitates the EMT, metastasis and invasion of CRC cells by obstructing the ubiquitination of the transcription factor SLUG, thereby inhibiting the ubiquitination and degradation of SLUG and enhancing the stability of SLUG protein (26).

#### 3.3.5 CircSKA3

When compared to the adjacent normal tissues, the expression level of circular RNA circSKA3 in colorectal cancer (CRC) tissues was notably elevated. Moreover, a high expression level of circular RNA circSKA3 was significantly associated with the tumor T stage, lymph node involvement status, the presence of distant metastasis, tumor size, and the histological grade of the tumor. Consistently, the silencing of circular RNA circSKA3 remarkably suppressed the proliferation and migration of CRC cells, and significantly promoted the apoptosis of these cells (27). As a pivotal regulator in the Wnt signal transduction process, β-catenin assumes a vital role in the Wnt signaling pathway and the development of colon cancer (52, 53). The activation of the Wnt/β-catenin signaling pathway enhances the viability, proliferation, and migratory capacity of CRC cells. The degradation of β-catenin predominantly occurs via its interaction with CK1, GSK3, andβ-TrCP, after which β-catenin is degraded by the ubiquitin-proteasome system (54). Song et al. is research validated the interaction between circular RNA circSKA3 and β-catenin. Circular RNA circSKA3 diminishes the interaction between β-catenin and the CK1/GSK3β/β-TrCP1 complex. It achieves this by directly binding to β-catenin, thereby inhibiting the proteasomal degradation of β-catenin. As a result, it reduces the degree of β-catenin ubiquitination and promotes an elevation in the β-catenin protein level within CRC cells (27). In summary, circular RNA circSKA3 is capable of facilitating the proliferation and migratory activity of CRC cells. It does so by suppressing the ubiquitination-mediated degradation of β-catenin and activating the Wnt/β-catenin signaling pathway (27).

### 3.4 Liver cancer

#### 3.4.1 Circ0006646

The expression level of circular RNA circ0006646 was notably upregulated in hepatocellular carcinoma (HCC) tissues. A high expression level of circular RNA circ0006646 was correlated with a higher TNM stage. Moreover, such high expression of circular RNA circ0006646 was indicative of a poor prognosis. Circular RNA circ0006646 has the ability to augment the metastatic potential of HCC cells both *in vitro* and *in vivo*. Nucleolin (NCL), one of the most abundantly expressed proteins within the nucleolus, has its upregulation potentially influencing the generation, proliferation, and metastatic ability of tumor cells by enhancing ribosomal RNA (rRNA) synthesis and the assembly of functional ribosomes, ultimately contributing to the advancement of cancer (55). The E3 ubiquitin ligase TRIM21 is capable of engaging in an interaction with NCL, which significantly elevates the level of ubiquitination of NCL (28). Circular RNA circ0006646 is also able to bind to NCL. The binding sites between circular RNA circ0006646 and NCL coincide with the binding sites of the E3 ubiquitin TRIM21 on NCL. As a consequence, this overlapping binding prevents the ubiquitination of NCL and stabilizes the expression level of NCL. The alteration in the expression level of NCL within HCC tissues can significantly influence the expression level of p53. NCL possesses the ability to bind to the 5’ untranslated region (UTR) of the mRNA encoding the tumor suppressor protein p53, thereby impeding the translation of p53. The tumor suppressor protein p53 plays a pivotal role in regulating crucial biological functions, including apoptosis, metabolic balance, and the immune system’s functionality. These functions are intricately linked to the onset and progression of tumors (56). In general, circular RNA circ0006646 has the capacity to maintain the stability of nucleolin (NCL) expression and modulate the metastasis of hepatocellular carcinoma (HCC) cells. It accomplishes this by obstructing the interaction between NCL and the E3 ubiquitin ligase TRIM21, thereby preventing NCL from being targeted for ubiquitination and degradation (28).

#### 3.4.2 CircDDX21

Glycolysis assumes a pivotal role in sustaining the viability and proliferation of cancer cells (57). In order to identify novel circRNAs involved in the regulation of glucose metabolism in HCC cells, Luo et al. detected circRNAs that were upregulated in HCC cells under glucose-deprived conditions. CircDDX21 is upregulated in HCC cells following glucose deprivation. It has been identified as a circRNA that is induced by glucose deprivation and is involved in the regulation of glucose metabolism (29). The specific mechanism is that circDDX21 promotes glycolysis through upregulating PGAM1 expression, thereby facilitating the *in-vivo* growth of hepatocellular carcinoma cells. Firstly, circDDX21 is capable of interacting with Polyadenylate-binding protein 1(PABPC1) and synergistically enhancing the stability of PGAM1 mRNA. Secondly, circDDX21 competes with MKRN3 for binding to PABPC1, thereby disrupting the interaction between MKRN3 and PABPC1 and inhibiting MKRN3-mediated ubiquitination of PABPC1. Unlike the ubiquitination-mediated degradation of other proteins, MKRN3-mediated ubiquitination of PABPC1 has no impact on the stability of PABPC1, yet it weakens the interaction between PABPC1 and its target mRNA (58). CircDDX21 enhances the interaction between PABPC1 and PGAM1 mRNA by inhibiting MKRN3-mediated ubiquitination of PABPC1, and upregulates the expression of PGAM1 protein to facilitate glycolysis. Therefore, circDDX21 exerts its tumor-promoting effect by regulating the ubiquitination of PABPC1 to elevate the level of PGAM1 protein in hepatocellular carcinoma (HCC) cells (29).

#### 3.4.3 CircFOXP

circFOXP is a circular RNA (circRNA) that is highly expressed in patients who have not experienced postoperative recurrence, and it is closely associated with a decreased incidence of postoperative intrahepatic cholangiocarcinoma (ICC) recurrence. When compared with patients who experienced ICC recurrence, the expression level of circFOXP1 in the tumors of patients who did not experience postoperative recurrence was significantly higher. Moreover, patients with a high expression level of circFOXP1 had a longer overall survival (OS) time and a lower recurrence rate. Correspondingly, the silencing of circFOXP1 significantly enhanced the proliferation, colony formation, and invasion abilities of intrahepatic cholangiocarcinoma (ICC) cells both *in vitro* and *in vivo* (30). Unlike other non-coding circular RNAs (circRNAs), circFOXP1 possesses protein-coding capacity and encodes a newly identified protein named circFOXP1-231aa. The expression of circFOXP1-231aa is capable of suppressing the proliferation, colony formation, and invasion of ICC cells (30). OTUD4 is a deubiquitinating protease that lowers the ubiquitination level of target proteins through the process of deubiquitination (59). The nuclear receptor co-activator 4 (NCOA4) plays a crucial role in the ferritin autophagy pathway. The autophagic degradation of ferritin is predominantly regulated by the intracellular concentration of NCOA4 (60). circFOXP1-231aa promotes the OTUD4-mediated deubiquitination of NCOA4 and decreases the ubiquitination level of NCOA4 by directly binding to OTUD4. As a consequence, it stabilizes the expression of NCOA4 and augments the process of ferroptosis. In conclusion, circFOXP1 inhibits the degradation of NCOA4 and potentiates ferroptosis by encoding the circFOXP1-231aa protein, which directly interacts with OTUD4, thereby exerting a tumor-suppressive function (30).

#### 3.4.4 CircCDYL

Cancer immunotherapy has achieved significant breakthroughs in cancer treatment, offering hope for the treatment of patients with advanced hepatocellular carcinoma (HCC), but immunotherapy resistance persists as a major obstacle to improving clinical outcomes (61). Fu et al. found that among cancer patients receiving anti-PD-L1 therapy, those with hepatocellular carcinoma (HCC) who had double-high expression of HRNR and CircCDYL were associated with elevated levels of CEA, ALT, and AST, as well as larger tumor diameters, compared to the non-double-positive group (31). It is suggested that there is a close relationship between the co-expression of HRNR and circCDYL and the efficacy of anti-PD-L1 immunotherapy. Previous studies have demonstrated that hornerin (HRNR)—a member of the S100 protein family—plays a role in regulating the mTOR signaling pathway and that it is a protein that promotes HCC tumor growth (62). SYVN1, which acts as an E3 ubiquitin ligase for HRNR, can interact with HRNR to enhance the latter’s ubiquitination level, thereby reducing HRNR expression (31). CircCDYL directly binds to HRNR, and precludes the binding of HRNR to its E3 ligase SYVN1, and ultimately inhibits HRNR ubiquitination and stabilizes HRNR protein expression. CircCDYL overexpression enhanced mTORC1 phosphorylation and augmented p70S6K activity. CircCDYL activates the mTOR pathway by binding to HRNR and stabilizing it. Previous studies have demonstrated that PD-L1 expression is regulated by the mTOR-p70S6K signaling pathway (63). CircCDYL upregulates PD-L1 expression, which may attenuate anti-PD-L1 therapy efficacy via PD-L1+ exosomes (64). In summary, CircCDYL stabilizes HRNR expression by directly binding to HRNR and preventing its ubiquitination, thereby activating the mTOR signaling pathway to upregulate PD-L1 expression in hepatocellular carcinoma (HCC) cells, ultimately leading to the reduction in the sensitivity of HCC cells to anti-PD-L1 therapy and the sustainment of tumor invasiveness (31).

#### 3.4.5 CircCCNY

Yang et al. aimed to identify differentially expressed circRNAs between parental HCC cells and lenvatinib-resistant tumor cells, revealing that circCCNY was significantly downregulated in the resistant cells (32). Furthermore, HCC patients with high circCCNY expression exhibited superior overall survival (OS) and recurrence-free survival (RFS). Consistently, circCCNY overexpression significantly suppressed HCC progression and restored lenvatinib sensitivity in HCC cells *in vitro*. Furthermore, both *in vitro* and *in vivo* experiments showed that circCCNY inhibition promoted lenvatinib resistance and induced immune evasion in HCC. Heat shock protein 60 (HSP60) participates in cell survival, apoptosis and the translocation of molecules between mitochondria and the cytoplasm, thus playing a pivotal role in signal transduction (65). HSP60 can upregulate the expression of c-Myc and PD-L1; notably, high PD-L1 expression is associated with compromised tumor immune function, an invasive disease phenotype, and poor prognosis in HCC patients (66). In addition, previous research has demonstrated that HSP60 interacts with RKIP (Raf kinase inhibitory protein) to regulate its function—thereby enabling HSP60 to also mediate activation of the MAPK signaling pathway via binding to RKIP (67). This interaction contributes significantly to HCC progression. E3 ubiquitin ligase SMAD ubiquitination regulatory factor 1 (SMURF1) is an E3 ubiquitin ligase for HSP60, and it can interact with HSP60 to enhance HSP60 ubiquitination. CircCCNY can interact with the HSP60 and SMURF1 protein complex and promote SMURF1-mediated HSP60 ubiquitination and subsequent degradation (32). Subsequently, HSP60 downregulation facilitated RKIP-mediated inactivation of the MAPK signaling pathway, thereby enhancing lenvatinib sensitivity and suppressing immune evasion in HCC. In addition, circCCNY downregulates PD-L1 expression and reduces immune evasion by inhibiting the HSP60/c-Myc signaling pathway. In summary, circCCNY regulates SMURF1-mediated HSP60 ubiquitination to reduce HSP60 protein levels in HCC cells, thereby inhibiting tumor immune evasion in HCC and enhancing sensitivity to lenvatinib treatment (32).

## 4 Cancers related to the female reproductive system

**Figure 3:**
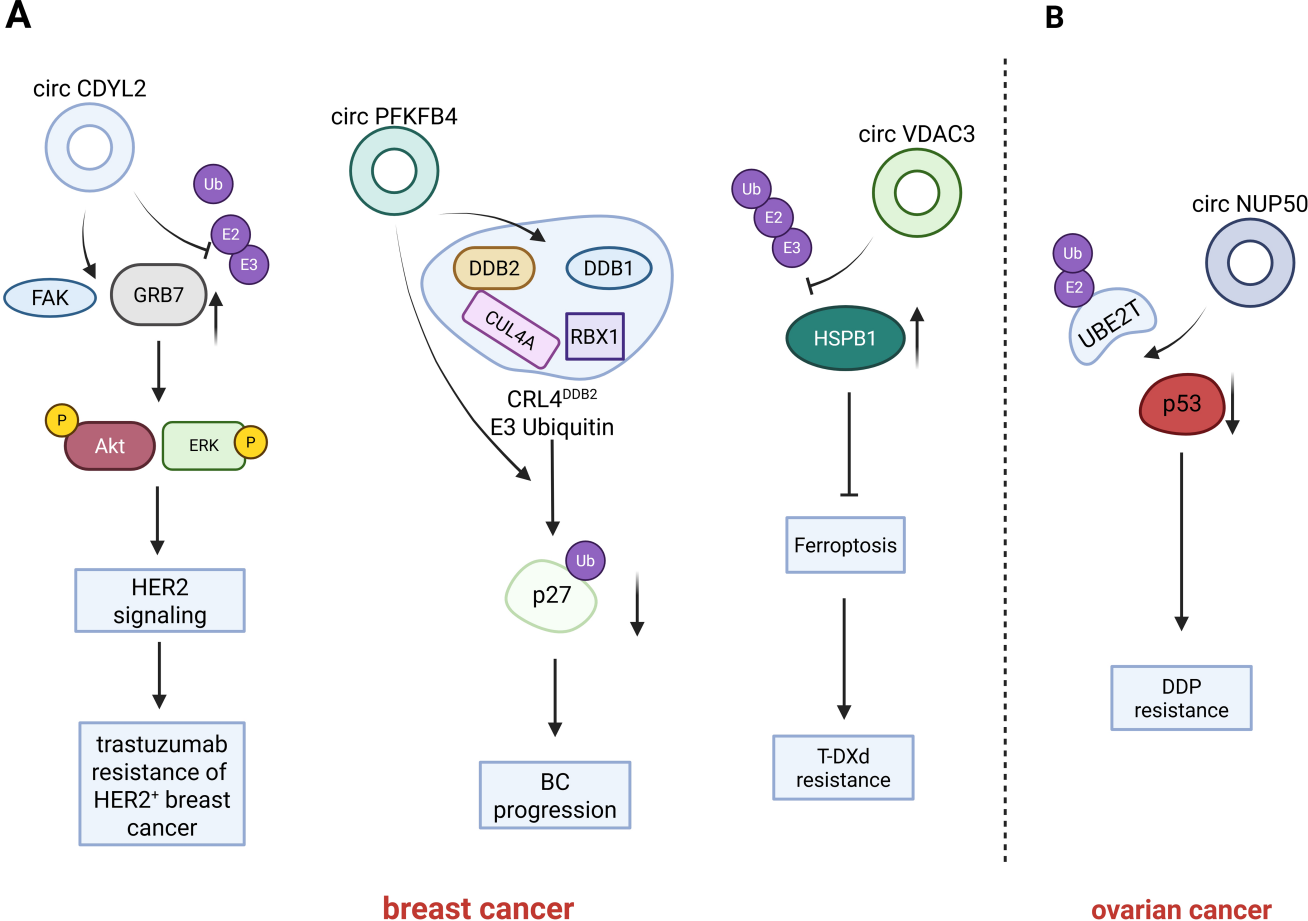
Introduction of circRNAs function on the breast cancer and ovarian cancer. circCDYL2 inhibiting the ubiquitination-mediated degradation of GRB7, and potentiates the interaction between GRB7 and FAK. Under hypoxic conditions, circPFKFB4 binds tightly to the CRL4DDB2 ubiquitin ligase complex, augments the interaction between DDB1 and DDB2, and prevents DDB2 degradation by the ubiquitin-proteasome system while further facilitating the assembly of the CRL4DDB2 ubiquitin ligase complex; circVDAC3 binds to HSPB1 in competition with ubiquitinating enzymes, preventing HSPB1 is ubiquitination-mediated degradation; CircNUP50 binds to p53 and UBE2T and promoting the ubiquitination-mediated degradation of p53.

Cancers related to the female reproductive system, such as breast cancer (BC) and ovarian cancer (OC), are notable examples. Breast cancer is the most prevalent malignancy among women (68), significantly threatening women’s health. It is of great significance to enhance early detection and screening measures for the effective control of breast cancer. Circular RNAs (circRNAs) possess the potential to serve as early biomarkers and therapeutic targets for breast cancer. Moreover, accumulating evidence from various studies indicates that circRNAs can influence the initiation and progression of breast cancer (69). (**Figure 3** and **Table 2**).

**Table 2:** Effects of circRNA on cancers related to the female reproductive system.

Disease	CircRNA	Protein ubiquitination level	Signaling pathways	Function	Ref
Breast carcinoma	CircCDYL2	Reduce the ubiquitination level of GRB7	HER2	Promote trastuzumab resistance in HER2 + breast cancer	(70)
	CircPFKFB4	Reduce the ubiquitination level of DDB2	p27	Promote the growth and metastasis of BC cells	(71)
	CircVDAC3	Reduce the ubiquitination level of HSPB1	Apoptosis	Mediating T-DXd resistance	(72)
Ovarian carcinoma	CircNUP50	Increase the ubiquitination level of p53	p53	Mediated platinum resistance in OC	(73)

### 4.1 Breast carcinoma

#### 4.1.1 CircCDYL2

Circular RNA CDYL2 (circCDYL2) is a circular RNA that exhibits high expression levels in breast cancer (BC) and is correlated with trastuzumab resistance in HER2-positive BC patients. When compared patients with low circCDYL2 expression, those with high circCDYL2 expression experienced more rapid relapse following anti-HER2 therapy. Additionally, patients with high circCDYL2 expression had significantly shorter disease-free survival (DFS) and overall survival (OS) periods. Consistently, the silencing of circCDYL2 significantly abrogated the trastuzumab resistance in BC cells and it significantly decreased the phosphorylation levels of AKT and ERK (70). Growth factor receptor-bound protein 7 (GRB7) is an adaptor protein that plays a role in the HER2 signaling pathway by triggering the phosphorylation of AKT and ERK, thereby facilitating cell survival and cell migration (74). Moreover, the degradation of GRB7 is exquisitely regulated by the Pin1-mediated ubiquitin-proteasome system (75).CircCDYL2 is capable of interacting with the GRB7 protein. Furthermore, upon the overexpression of circCDYL2, the protein expression level of GRB7 is elevated. circCDYL2 inhibits the binding of Pin1 with the GRB7 by interacting with GRB7, which leads to a decrease in the ubiquitination level of GRB7 and thereby stabilizes the expression of GRB7, resulting in the up-regulation of the GRB7 protein expression in HER2-positive breast cancer cells. In addition, the sustained activation of the AKT and ERK signaling pathways necessitates the interaction between FAK and GRB7 for its maintenance (76). CircCDYL2 is able to form a complex with GRB7 and FAK proteins. Specifically, circCDYL2 functions as a scaffold molecule, mediating the protein-protein interaction, which in turn augments the interaction between GRB7 and FAK (70). In conclusion, circCDYL2 upregulates the expression of GRB7 by inhibiting the ubiquitination-mediated degradation of GRB7. Additionally, it potentiates the interaction between GRB7 and FAK, which sustains the activity of the downstream signaling molecules, AKT and ERK. Consequently, these actions of circCDYL2 contribute to the development of trastuzumab resistance in HER2+ breast cancer (70).

#### 4.1.2 CircPFKFB4

Chen et al. identified a novel circular RNA (circRNA), circPFKFB4, through the comparison of differentially expressed circRNAs in breast cancer cells under normoxic and hypoxic conditions. When compared to breast cancer (BC) cells cultured under normoxic conditions, circular RNA PFKFB4 (circPFKFB4) demonstrated the most substantial alteration in expression levels and was up-regulated in hypoxic cells. Moreover, the expression of circPFKFB4 in BC tissues was significantly elevated when compared to that in matched normal breast tissues. Additionally, its overexpression was positively associated with the TNM stage and poor prognosis in BC patients (71). In line with previous findings, circPFKFB4 promotes the proliferation of hypoxic BC cells both *in vitro* and *in vivo* (71). The CRL4DDB2 ubiquitin ligase complex is a heterodimeric complex composed of DDB1 and DDB2, which further interacts with CUL4A and RBX1 to form the complete complex (77), its function is related to the ubiquitination and degradation of p27 (78). CircPFKFB4 is capable of directly binding to DNA damage-binding protein 2(DDB2). In hypoxic BC cells, it prevents the degradation of DDB2 by the ubiquitin-proteasome system, thereby enhancing the protein expression level of DDB2. Under hypoxic conditions, circPFKFB4 augmented the interaction between DDB1 and DDB2 within BC cells, and circPFKFB4, DDB1, and DDB2 formed trimers circPFKFB4 binds tightly to the CRL4^DDB2^ ubiquitin ligase complex, and it further facilitates the assembly of the CRL4^DDB2^ ubiquitin ligase complex Under hypoxic conditions. As a tumor suppressor protein, p27 is capable of inhibiting the expression of CDKs and inducing cell cycle arrest (79). CircPFKFB4 also enhanced the recognition and binding of the CRL4DDB2 ubiquitin ligase complex for p27; and thus, circPFKFB4 promotes the CRL4DDB2 ubiquitin ligase-mediated ubiquitination and subsequent degradation of p27 through multiple mechanisms. In summary, under hypoxic conditions, the hypoxia-induced circPFKFB4 exerts a tumor-promoting function by facilitating the CRL4DDB2 ubiquitin ligase-mediated degradation of p27, consequently accelerating the progression of BC (71).

#### 4.1.3 CircVDAC3

Zou et al. screened for circular RNAs (circRNAs) that were highly expressed in HER2-low-expression breast cancer cells and determined that circVDAC3 had the highest expression level among them in HER2-low-expression breast cancer cells *in vivo* (72). Overexpression of circular RNA VDAC3 (circVDAC3) significantly decreased the sensitivity of breast cancer cells to trastuzumab deruxtecan (T-DXD) treatment. This finding indicates that circVDAC3 is associated with trastuzumab deruxtecan resistance in HER2-low-expression breast cancer. The specific mechanism is that circVDAC3 and heat-shock protein beta-1(HSPB1) are capable of binding to each other. The binding domain of circVDAC3 to HSPB1 encompasses multiple lysine ubiquitination sites. By binding to HSPB1, circVDAC3 impedes the ubiquitination of HSPB1, thereby elevating the level of HSPB1 protein (72). Previous investigations have demonstrated that elevated expression of HSPB1 is capable of inhibiting ferroptosis (80, 81). Zou et al. discovered that the sensitivity of circVDAC3-overexpressing cells to trastuzumab deruxtecan (T-DXD) treatment could be significantly restored by directly inducing ferroptosis. This finding indicates that trastuzumab deruxtecan resistance in breast cancer is associated with HSPB1-mediated inhibition of ferroptosis. In conclusion, circVDAC3 mediates trastuzumab deruxtecan (T-DXd) resistance in breast cancer by competitively binding to HSPB1 in competition with ubiquitinating enzymes, which prevents the ubiquitination-mediated degradation of HSPB1, upregulates the expression of HSPB1 protein, and consequently inhibits ferroptosis (72).

### 4.2 Ovarian carcinoma

#### 4.2.1 CircNUP50

Zhu et al. conducted a screening to identify circRNAs showing differential expression in platinum-resistant OC tissues in comparison with PS ovarian cancer tissues. Compared with matched platinum-sensitive tissues and cells, circNUP50 was determined to be overexpressed in platinum-resistant OC tissues and cells (73). Ubiquitin-conjugating enzyme E2T (UBE2T) is a crucial ubiquitin-conjugating enzyme among the binding proteins involved in the ubiquitination process. It promotes cancer chemoresistance by facilitating RING1-mediated ubiquitination and degradation of p53 (82). CircNUP50 is capable of simultaneously binding to p53 and UBE2T. By acting as a scaffold, circNUP50 promotes the interaction between p53 and UBE2T, enhancing p53 ubiquitination and consequently reducing p53 expression. Furthermore, circNUP50 functions as a miR-197-3p sponge, resulting in the up-regulation of Ras GTPase-activating protein-binding protein 1(G3BP1) protein expression, When G3BP1 interacts with ubiquitin-specific protease 10 (USP10), it impedes the interaction between USP10 and p53, consequently leading to the inhibition of p53 deubiquitination (83). In conclusion, circNUP50 mediates platinum resistance in OC through two distinct mechanisms: it both directly regulates p53 ubiquitination and, by acting as a sponge for miR-197-3p, indirectly promotes the degradation of ubiquitinated p53, ultimately leading to impacts on the cell cycle and reduction in apoptosis (73).

## 5 Urogenital neoplasms

**Figure 4:**
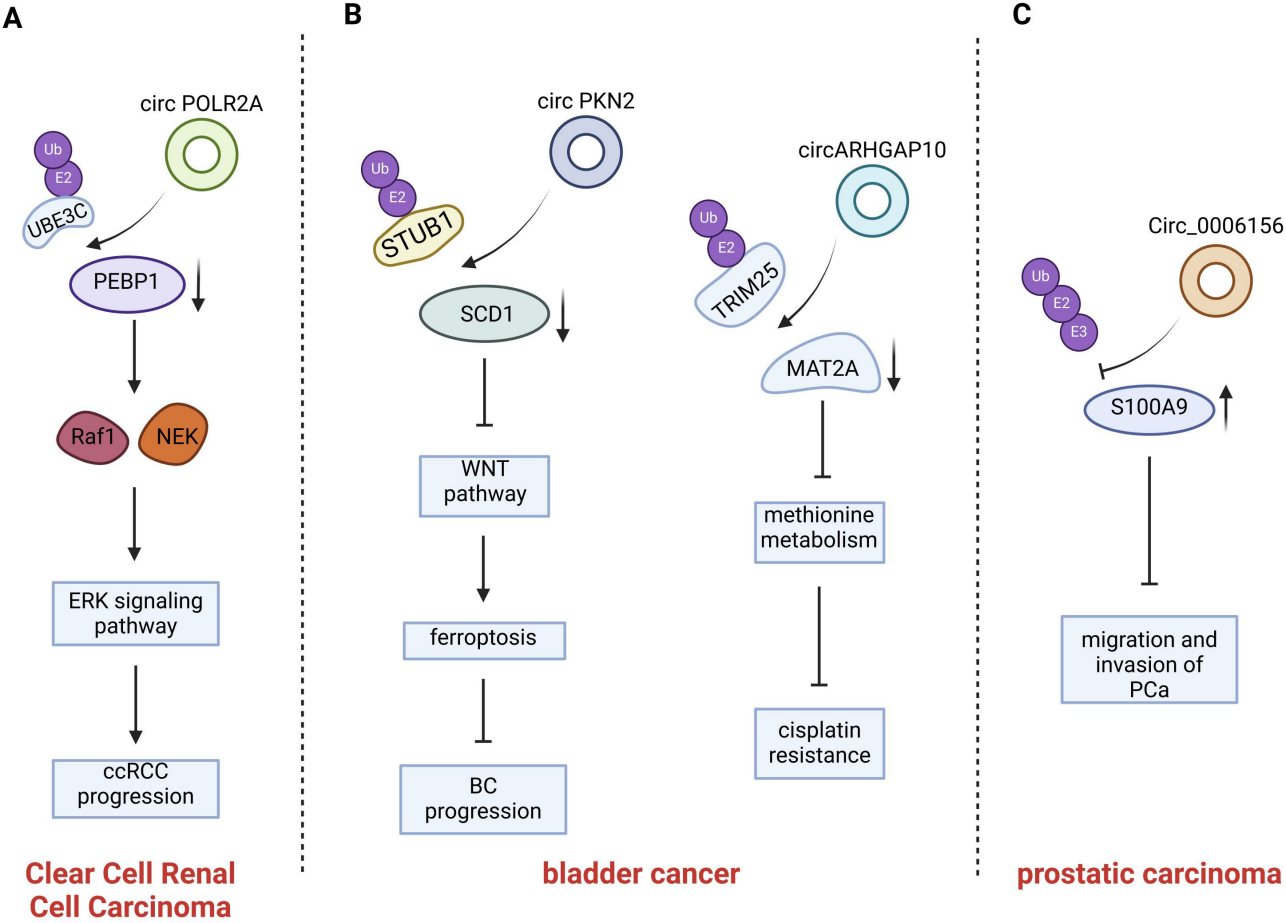
Introduction of circRNAs function on the urogenital neoplasms. CircPOLR2A directly binds PEBP1 and UBE3C and accelerates ubiquitination-mediated PEBP1 degradation; CircPKN2 binds to the SCD1 protein, recruits the E3 ubiquitin ligase STUB1 to interact with SCD1 and promotes ubiquitination-mediated SCD1 degradation; CircARHGAP10 mediates TRIM25-MAT2A interaction, promoting MAT2A ubiquitination; Circ0006156 binds to the S100A9 protein and prevents E-3 ubiquitin-protein ligases from binding to the S100A9 protein.

Renal carcinoma, prostate carcinoma, and bladder carcinoma are the most prevalent tumors of the urinary system. Early-stage urinary system tumors can often be effectively treated and can be cured through surgical intervention. However, advanced-stage urinary system tumors, which are usually incurable, necessitate comprehensive systemic treatment approaches, which typically encompass targeted therapy and immunotherapy. Consequently, it is of utmost significance to identify early diagnostic biomarkers and potential therapeutic targets for the diagnosis and treatment of urinary system malignancies. Currently, there is substantial evidence indicating that the aberrant expression of circular RNAs (circRNAs) within the body is strongly correlated with the development of urinary system malignancies (84). It provides new ideas and strategies for the diagnosis and precision-targeted therapy of urinary system malignancies (85). (**Figure 4** and **Table 3**).

**Table 3:** Effects of circRNA on urogenital neoplasms.

Disease	CircRNA	Protein ubiquitination level	Signaling pathways	Function	Ref
Renal carcinoma	CircPOLR2A	Increase the ubiquitination level of PEBP1	Raf1/MEK/ERK	Promotes ccRCC proliferation, migration, invasion and angiogenesis, and inhibits apoptosis	(86)
Bladder carcinoma	CircPKN2	Increase the ubiquitination level of SCD1	SCD1/WNT	Inhibit the proliferation, invasion and migration of BC cells	(87)
	CircARHGAP10	Increase the ubiquitination level of MAT2A	methionine metabolism	Reduce the cisplatin resistance of BCa cells	(88)
Prostate carcinoma	Circ0006156	Reduce the ubiquitination level of S100A9	S100A9	Inhibit the migration and invasion of PCa cell	(89)

### 5.1 Renal carcinoma

#### 5.1.1 CircPOLR2A

Xu et al. found that circPOLR2A showed elevated expression in the cancerous tissues of ccRCC compared with the matched adjacent normal tissues. Moreover, Xu et al. found that the upregulation of circPOLR2A was significantly correlated with tumor size and TNM stage in cRCC patients (86). Phosphatidylethanolamine Binding Protein 1 (PEBP1) is a member of the phosphatidylethanolamine-binding protein family, PEBP1 is able to competitively bind to Raf1, which results in the dissociation of the Raf1-MEK complex and directly inhibits the activation of Raf-1. As a result, PEBP1 functions as an inhibitor of the Raf1/MEK/ERK signaling pathway (90). In addition, PEBP1 is also involved in regulating cell proliferation, mitosis and drug-induced apoptosis. Consequently, PEBP1 can be considered as a suppressing factor of cancerous progression. CircPOLR2A functions as a cancer-promoting factor in cRCC. It facilitates cell proliferation, migration, invasion, and angiogenesis while suppressing apoptosis. As a ubiquitin E3 ligase, UBE3C plays a role in the degradation of the PEBP1 protein within ccRCC cells via the ubiquitin-proteasome system (86).CircPOLR2A can directly bind to PEBP1 and UBE3C. It functions as a scaffold between PEBP1 and UBE3C, thereby promoting their interaction and accelerating the ubiquitination-mediated degradation of PEBP1. In conclusion, circPOLR2A influences the proliferation and metastasis of cRCC through the regulation of PEBP1 ubiquitination, thereby playing a role in cancer promotion (86).

### 5.2 Bladder carcinoma

#### 5.2.1 CircPKN2

circPKN2, a circular RNA, exhibits significantly decreased expression in bladder cancer (BC) cells and is associated with the proliferation, migration, invasion, and epithelial-mesenchymal transition (EMT) process of BC cells. BC patients with high circPKN2 expression had better overall survival (OS) and disease-free survival (DFS) as compared to patients with low expression. There was a correlation between low-expressed circPKN2 and the clinical characteristics of BC patients, with associations in tumor size, N stage, and muscle invasion. In line with this, Liu et al. found that both the tumor volumes and weights of mice were significantly decreased in the circPKN2-overexpression group compared with circPKN2 knockdown group. And in the circPKN2 knockdown group, tumors of mice showed increased SCD1, SLC7A11, N-cadherin, and Vimentin expression and decreased TFR1 expression, while circPKN2 overexpression led to opposite changes. These results imply that circPKN2 promotes ferroptosis in BC cells *in vivo*, inhibits its proliferation, and suppresses the EMT (87). The specific mechanism is that Stearoyl-CoA desaturase 1 (SCD1) has been verified to activate the WNT pathway and to regulate the ferroptosis process of various cell types, thereby exerting a pro-carcinogenic function (91, 92), but CircPKN2 can directly bind to SCD1. When circPKN2 binds to the SCD1 protein, it recruits the E3 ubiquitin ligase STUB1 to interact with SCD1 and significantly strengthens the interaction between SCD1 and STUB1, thus promoting the ubiquitination-mediated degradation of SCD1 (87). Collectively, circPKN2 affects the SCD1/WNT signaling pathway by regulating the ubiquitination-mediated degradation of SCD1. Consequently, it promotes ferroptosis in BC cells to inhibit the proliferation, invasion, and migration of BC cells (87).

#### 5.2.2 CircARHGAP10

As one of the essential amino acids, methionine is involved in methionine metabolism that provides a one-carbon unit through the methyl donor S-adenosylmethionine (SAM). This process is crucial for maintaining protein metabolic homeostasis, which sustains cell growth (93). Methionine, for example, participates in activities necessary to support purine biosynthesis, thymidine biosynthesis, redox balance and epigenetic regulation (94). Methionine adenosine transferase (MATS) is a key enzyme in the initiation of methionine metabolism (95). Yang et al. found that Methionine adenosyl transferase IIa (MAT2A) was highly expressed in Bca cells. They also discovered that the combination of methionine restriction and chemotherapy achieved favorable outcomes compared to chemotherapy alone. These findings indicate that methionine metabolism is involved in cisplatin resistance in BCa cells (88). Since MAT2A is extensively expressed in diverse tissues, it is difficult to directly target MAT2A to overcome the cisplatin resistance of BCa caused by MAT2A-mediated methionine metabolism. Yang et al. performed high-throughput circRNA Sequencing on cisplatin-resistant BCa cells and normal BCa cells as well as on five pairs of benign and malignant bladder tissue to identify differentially expressed circRNAs. CircARGHAP10 was identified as a circular RNA associated with methionine metabolism. Notably, the expression of circARGHAP10 was downregulated in bladder cancer tissue when compared with the paired normal bladder tissue (88). CircARHGAP10 has the potential to overcome cisplatin resistance in BCa cells by promoting the ubiquitination-mediated degradation of MAT2 A. TRIM25 as an E3 ligase interacts with MAT2A. This interaction can enhance the ubiquitination levels of MAT2A and facilitate the degradation of MAT2A (88). circARHGAP10 functions as an RNA scaffold to mediate the direct interaction between TRIM25 and MAT2A, thereby promoting TRIM25-dependent ubiquitination of MAT2A (88). Since MAT2A-mediated methionine metabolism is critical for the cisplatin resistance phenotype of BCa cells, the inhibition of MAT2A by circARHGAP10 over-expression and the restriction of methionine uptake were sufficient to overcome cisplatin resistance *in vivo* in an immuno- deficiency model. However, the approach could not achieve similar *in vivo* efficacy against cisplatin-resistant BCa cells within an immunocompetent model. Overcoming cisplatin resistance in BCa cells within an immunocompetent model necessitates a combination therapy that includes inhibiting MAT2A-regulated methionine metabolism and alleviating SLC7A6-mediated CD8+ T cell dysfunction. However, immune-related issues are not covered in this review. In summary, circARHGAP10 modulates methionine metabolism-mediated cisplatin resistance through the regulation of MAT2A ubiquitination processes and elicits its anti-tumor effect (88).

### 5.3 Prostate carcinoma

#### 5.3.1 Circ0006156

Zhang et al. aimed to investigate differentially expressed circRNAs in prostate cancer (PCa) tissue. They conducted high-throughput circRNA sequencing on two pairs of PCa and matched adjacent non-tumor tissue samples and discovered that circ0006156 was expressed at low levels in PCa tissues. It was also ascertained that the protein expression of Protein S100-A9(S100A9) was positively regulated by the overexpression of circ0006156, and that circ0006156 was negatively correlated with the aggressive phenotypes of PCa cells (89). Consistently, circ0006156 suppresses PCa cell metastasis by stabilizing S100A9 in an *in-vivo* setting. The S100A9 protein is a member of the S100 protein family. Numerous prior investigations have demonstrated that the expression level of S100A9 in PCa tissues is significantly lower than that in benign prostatic hyperplasia tissues, thereby implying that the S100A9 protein serves as a suppressor of prostate cancer progression (96). Circ0006156 binds to and stabilizes S100A9 by obstructing ubiquitination. The specific mechanism is that circ0006156 binds to the S100A9 protein and prevents E-3 ubiquitin-protein ligases from binding to the S100A9 protein. This action reduces the degradation of the S100A9 protein, thereby inhibiting the migration and invasion of PCa cells (89). After silencing of circ0006156, the overexpression of the S100A9 protein is also capable of inhibiting the migration and invasion of PCa cells. These findings unequivocally demonstrate that circ0006156 functions as a tumor suppressor by modulating the ubiquitination process of the S100A9 protein and maintaining the stability of S100A9 protein expression, thereby effectively inhibiting the migration and invasion of PCa cells (89).

## 6 Cancers of other systems

**Figure 5:**
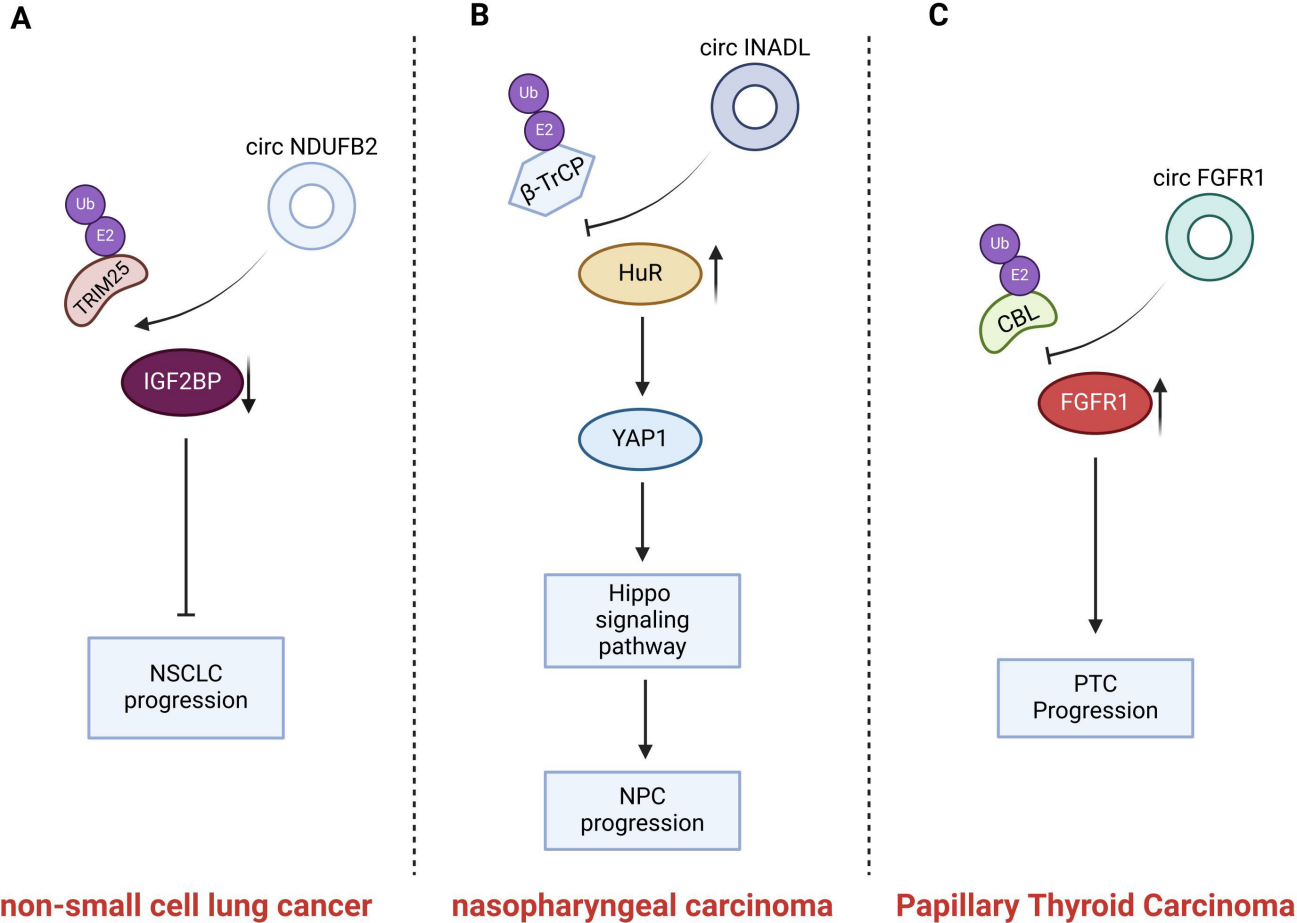
Introduction of circRNAs function on the non-small cell lung cancer and nasopharyngeal carcinoma and thyroid carcinoma. CircNDUFB2 mediates TRIM25- IGF2BP interaction, promoting IGF2BP ubiquitination; CircINADL binds HuR, impeding β-TrCP and HuR interaction and inhibiting HuR ubiquitination and degradation; circFGFR1 blocks FGFR1 protein ubiquitination, reducing its degradation.

There is mounting evidence suggesting that certain tumors within other systems also exhibit abnormal expression of circular RNAs (circRNAs). A plethora of circular RNAs (circRNAs) assume a pivotal role in the process of cancer cells progression by modulating the stability of oncogenic or tumor suppressor proteins within cancer cells. (**Figure 5** and **Table 4**).

**Table 4:** Effects of circRNA on cancers of other systems.

Disease	CircRNA	Protein ubiquitination level	Signaling pathways	Function	Ref
Non-small cell lung carcinoma	CircNDUFB2	Increase the ubiquitination level of igf2bp	RNA-binding protein	Inhibit the proliferation and metastasis of NSCLC	(39)
Nasopharyngeal carcinoma	CircINADL	Reduce the ubiquitination level of HuR	Hippo	Promote the metastasis and EMT of nasopharyngeal carcinoma cells	(97)
Thyroid carcinoma	CircFGFR1	Reduce the ubiquitination level of FGFR1	FGFR1	Promote PTC cell proliferation and inhibit apoptosis	(98)

### 6.1 Non-small cell lung carcinoma

#### 6.1.1 CircNDUFB2

CircNDUFB2 is derived from exons 2–3 of the NDUFB2 gene and has a length of 249 nt. Li et al. identified the differential expression of the dysregulated circRNAs in non-small cell lung cancer (NSCLC) samples using quantitative reverse transcription PCR (qRT-PCR). Among these dysregulated circRNAs, circNDUFB2 was the most significantly downregulated one (39). A high expression level of circNDUFB2 was negatively correlated with tumor size, lymph node metastasis status, and clinical stage in patients with NSCLC (39). Insulin-like growth factor 2 m RNA-binding protein (IGF2BP) belongs to an evolutionarily conserved RNA-binding carcinoembryonic protein family (99). The expression of IGF2BP is upregulated in NSCLC. Moreover, the overexpression of IGF2BP facilitates tumor growth and metastasis and is indicative of a poor prognosis (100). IGF2BP has the ability to promote cancer progression. However, circNDUFB2 is capable of decreasing the stability of IGF2BP and inducing its degradation. Li et al. demonstrated that circNDUFB2 interacts with IGF2BP proteins and promotes the degradation of these proteins via the ubiquitin-proteasome system in NSCLC cells. TRIM25 is the E3 ubiquitin ligase that catalyzes the ubiquitination of IGF2BPs.It precisely regulates the ubiquitination-dependent degradation of IGF2BPs. CircNDUFB2 serves as a scaffold, thereby enhancing the interaction between TRIM25 and IGF2BPs.Consequently, it facilitates the TRIM25-mediated ubiquitination and subsequent degradation of IGF2BPs (39). In summary, circNDUFB2 inhibits the proliferation and metastatic spread of NSCLC by facilitating the TRIM25-mediated ubiquitination and subsequent degradation of IGF2BPs. In addition to promoting the degradation of IGF2BPs, circNDUFB2 is also capable of triggering an immune response in NSCLC cells. CircNDUFB2 could potentially activate retinoic acid-inducible gene I (RIG-I). It achieves this by interfering with the intramolecular interactions between the caspase-activation and recruitment domains (CARDs) and the helicase domain of RIG-I. As a result, RIG-I is kept in an active state, which then sets off the activation of the RIG-I-MAVS signaling cascade (39). CircNDUFB2 impedes the progression of tumors via the activation of an immune response. However, the specific aspects pertaining to immunization have not been addressed in this review.

### 6.2 Nasopharyngeal carcinoma

#### 6.2.1 CircINADL

Circular RNA INADL (circINADL) was expressed at higher levels in tissue samples from nasopharyngeal carcinoma (NPC) patients compared to those from normal nasopharyngeal epithelial tissue samples. Moreover, a significant positive correlation was observed between circINADL and the clinical stage of NPC patients. Moreover, CircINADL can contribute to the promotion of migration, invasion, and EMT processes in NPC cells (97). Consistently, circINADL has the capacity to facilitate the metastasis of NPC cells *in vivo*. Human antigen R (HuR), an RNA-binding protein, plays a role in stabilizing target mRNA, thereby enhancing the stability of the mRNA and improving its translation efficiency (101). Yes-associated protein 1(YAP1) serves as a vital downstream mediator within the Hippo signaling pathway. Functioning in the role of a transcriptional co-activator, it facilitates the proliferation and survival of NPC cells (102). Previous investigations have revealed that β-transducin repeat-containing protein (β-TrCP) serves as an E3 ubiquitin ligase that specifically modulates the ubiquitination and subsequent degradation of HuR (103). Zhang et al. found that the expression of circINADL was able to decrease the ubiquitination level of HuR. The specific mechanism is that circINADL has the capacity to bind directly to HuR, thereby impeding the interaction between β-TrCP and HuR and subsequently inhibiting the ubiquitination and degradation processes of HuR (97). Subsequently, HuR exhibits the ability to bind to the 3 ‘-UTR of YAP1 Mrna, thereby facilitating the expression of YAP1. In summary, these findings demonstrate that circRNADL is capable of modulating the ubiquitination of HuR, thereby enhancing its expression in NPC cells. Moreover, circINADL promotes the metastasis and EMT of NPC cells through a mechanism in which HuR upregulates the expression of YAP1 and activates the Hippo signaling pathway (97).

### 6.3 Thyroid carcinoma

#### 6.3.1 CircFGFR1

Previous literature studies have reported the differential expression of circRNAs in PTC tissues compared with benign thyroid tissues (104). On this basis, Zheng et al. focused their research on circ0008016 (called circFGFR1), which was significantly up-regulated in PTC tissues. They discovered that circFGFR1 promoted PTC cell proliferation and inhibited apoptosis, thereby contributing to the progression of cancer (98). CBL is a crucial member of the E3 ubiquitin-ligase family, and it mediates the ubiquitination of Fibroblast growth factor receptor 1(FGFR1). CircFGFR1 is capable of interacting with CBL and obstructing the binding between CBL and FGFR1. Consequently, it inhibits CBL-mediated ubiquitination of FGFR1 in PTC cells and reduces the ubiquitination and degradation of the FGFR1 protein (98). The increased expression of FGFR1 protein is capable of promoting the migration and invasion of PTC cells (105). Furthermore, the inhibitory effects on cell proliferation and the induction of cell apoptosis caused by the silencing of circFGFR1 could be counteracted by the elevated expression of FGFR1. All these indicate that the upregulation of FGFR1 protein expression will promote the proliferation of PTC cells. In summary, circFGFR1 decreases the degradation of the FGFR1 protein by blocking the ubiquitination of the FGFR1 protein, thereby stabilizing the expression of the FGFR1 protein. It subsequently promotes the proliferation of PTC cells and inhibits apoptosis, ultimately contributing to its role in promoting cancer development (98).

## 7 Limitations and future perspectives

An increasing body of evidence indicates that circular RNAs (circRNAs) that are engaged in the precise regulation of protein stability assume a crucial role in the progression of malignant neoplasms. There are two main consequences regarding how circRNAs regulate the stability of target proteins.

1. CircRNAs promote the ubiquitination and degradation of target proteins. Currently, numerous studies have demonstrated that circRNAs are involved in promoting the degradation of key proteins in malignant tumors via multiple mechanisms. Firstly, circRNAs can directly bind to target proteins and E3 ligases, functioning as protein-protein scaffolds, which enhances the activity of E3 ligases and facilitates the ubiquitination of target proteins. Examples of such circRNAs include circNUP50, circPOLR2A, circPKN2, circARHGAP10, circNDUFB2, and circPFKFB4. Secondly, certain circRNAs are capable of encoding proteins that indirectly facilitate the ubiquitination of target proteins. For instance, circRACK1 and circPLCE1 are examples of such circRNAs. (**Table 5**).

**Table 5:** CircRNA-ubiquitin crosstalk.

circRNA-ubiquitin crosstalk	CircRNA	Protein ubiquitination level	Signaling pathways	Function	Ref
circRNA as scaffold	circSEC24B	Reduce the ubiquitination level of SRPX2	FAK/SRC/ERK	Promote the proliferation of CRC cells and activate autophagy to induce chemotherapy resistance	(24)
	circPFKFB4	Reduce the ubiquitination level of DDB2	p27	Promote the growth and metastasis of BC cells	(64)
	circNUP50	Increase the ubiquitination level of p53	p53	Mediated platinum resistance in OC	(66)
	circPOLR2A	Increase the ubiquitination level of PEBP1	Raf1/MEK/ERK	Promotes ccRCC proliferation, migration, invasion and angiogenesis, and inhibits apoptosis	(79)
	circPKN2	Increase the ubiquitination level of SCD1	SCD1/WNT	Inhibit the proliferation, invasion and migration of BC cells	(80)
	CircARHGAP10	Increase the ubiquitination level of MAT2A	methionine metabolism	Reduce the cisplatin resistance of BCa cells	(81)
	circNDUFB2	Increase the ubiquitination level of igf2bp	RNA-binding protein	Inhibit the proliferation and metastasis of NSCLC	(39)
	circCCNY	Increase the ubiquitination level of HSP60	MAPK and HSP60/c-myc/PD-L1	Inhibiting tumor immune escape in HCC and enhancing sensitivity to lenvatinib treatment	(32)
circRNA as decoy	circ0026611	Reduce the ubiquitination level of PROX1	lymphatic transcription factors	Enhance the migration, invasion and lymphangiogenesis of human lymphatic endothelial cells	(20)
	circNFATC3	Reduce the ubiquitination level of IGF2BP3	Non-coding RNA	Promoting tumor progression	(21)
	circ0124554	Reduce the ubiquitination level of AKT	AKT	Promote tumor cell proliferation and inhibit apoptosis	(23)
	circSKA3	Reduce the ubiquitination level of SLUG	EMT	Promote EMT, metastasis and invasion of CRC cells	(26)
	circSKA3	Reduce the ubiquitination level of β-catenin	Wnt/β-catenin	Promote the proliferation and migration of CRC cells	(27)
	circ0006646	Reduce the ubiquitination level of NCL	p53	Enhance the metastasis of HCC	(28)
	circDDX21	Reduce the ubiquitination level of PABPC1	glycolysis	Promoting the growth of hepatocellular carcinoma cells *in vivo*	(29)
	circCDYL2	Reduce the ubiquitination level of GRB7	HER2	Promote trastuzumab resistance in HER2 + breast cancer	(63)
	circVDAC3	Reduce the ubiquitination level of HSPB1	Apoptosis	Mediating T-DXd resistance	(65)
	circ0006156	Reduce the ubiquitination level of S100A9	S100A9	Inhibit the migration and invasion of PCa cell	(82)
	circINADL	Reduce the ubiquitination level of HuR	Hippo	Promote the metastasis and EMT of nasopharyngeal carcinoma cells	(90)
	circFGFR1	Reduce the ubiquitination level of FGFR1	FGFR1	Promote PTC cell proliferation and inhibit apoptosis	(91)
	circCDYL	Reduce the ubiquitination level of HRNR	mTOR-p70S6K	Reducing the sensitivity of HCC cells to anti-PD-L1 therapy and sustaining tumor invasiveness	(31)
circRNA encoded peptides as effectors	circPDE5A	Reduce the ubiquitination level of PIK3IP1	PI3K/AKT	Inhibit tumor proliferation and metastasis	(19)
	circRACK1	Increase the ubiquitination level of vimentin	EMT	Inhibition of gastric cancer cell metastasis	(22)
	circPLCE1	Increase the ubiquitination level of RPS3	NF-κB	Inhibit the proliferation and metastasis of CRC cells	(25)
	circFOXP	Reduce the ubiquitination level of NCOA4	Apoptosis	Inhibit the proliferation, colony formation and invasion of ICC cells	(30)

2. CircRNAs inhibit the ubiquitination and degradation of target proteins. The first mechanism is the most prevalent one. CircRNAs and E3 ubiquitin ligases share the same binding site on the target protein. This enables circRNAs to competitively inhibit the binding of E3 ubiquitin ligases, thereby decreasing the ubiquitination level of the target protein. Examples of such circRNAs include circ0026611, circNFATC3, circ0124554, circSKA3, circ0006646, circDDX21, circCDYL2, circVDAC3, circ0006156, circINADL, and circFGFR1. Secondly, circRNAs function as protein-protein scaffolds, which promotes the interaction between deubiquitinating enzymes and target proteins. By enhancing the activity of deubiquitinating enzymes, they reduce the ubiquitination level of target proteins. An example of such a circRNA is circSEC24B. Thirdly, certain circRNAs are able to indirectly facilitate the deubiquitination of target proteins by encoding specific proteins. A case in point is circPDE5A (**Table 5**).

The dysregulated expression of circRNAs is evident in numerous cancer types. As non-coding RNAs, dysregulated circRNAs exert regulatory influences on a wide array of tumor biological processes. These processes encompass sustaining proliferative signal transduction, aiding in cell migration and invasion, inhibiting apoptosis, and triggering angiogenesis. In addition, circRNAs are frequently related to the development of immunotherapy resistance in cancer. Specifically, in cancer immunotherapy, circRNAs regulate the expression of key proteins via ubiquitination modulation, thereby affecting the efficacy of cancer immunotherapy. For instance, circCDYL stabilizes HRNR expression to promote the secretion of PD-L1-positive exosomes and upregulate PD-L1 levels in tumor cells, thereby inducing resistance to anti-PD-L1 immunotherapy. Conversely, circCCNY inhibits the HSP60/c-Myc signaling pathway (via SMURF1-mediated HSP60 ubiquitination and degradation), thereby downregulating PD-L1 expression to inhibit tumor immune escape and improve the sensitivity to immunotherapy. CircRNAs are also involved in the regulation of tumor immunity. RIG-I and IRF7 are critical components of the immune signaling transmission mediated by circNDUFB2, and the induction of RIG-I and IRF7 by circNDUFB2 may constitute a positive feedback loop and provoke cellular immune responses, which can inhibit the progression of NSCLC. However, the causes of circRNA dysregulation and the mechanisms by which circRNAs exert their regulatory functions remain unclear. Future research should aim to identify the factors contributing to circRNA dysregulation, as well as the crucial structural elements or RNA sequences through which circRNAs exert their effects. Moreover, due to the stable expression of circRNAs in diverse tissues and their dysregulation in cancerous tissues, circRNAs have the potential to be promising biomarkers for cancer screening and prognosis. The potential of circRNAs as therapeutic targets is exemplified by the work of Zhu et al, who prepared platinum and si-circNUP50 codelivery nanosystems. These nanosystems effectively circumvent platinum resistance in ovarian cancer (OC) tumor models, thereby underscoring the crucial role of circNUP50 in platinum-resistant ovarian cancer. This approach, utilizing si-circNUP50, may offer a promising strategy for treating platinum-resistant ovarian cancer. And specific antisense oligonucleotides (ASOs) targeting circSKA3 can inhibit colorectal cancer (CRC) epithelial-mesenchymal transition (EMT) and metastasis by either inhibiting circSKA3 circularization or disrupting the interaction between circSKA3 and Slug. These ASOs have the potential to serve as novel therapeutic agents for cancer in the future. And the injection of sh-circ0006646 lentivirus has been demonstrated to decrease the metastatic potential of hepatocellular carcinoma (HCC) cells *in vivo*. These examples illustrate that circRNAs are anticipated to exert a profound influence on oncological treatments in the future. However, solely relying on circRNA-based therapy may not attain the objective of cancer treatment. For instance, the knockout of circARHGAP10 and the restriction of methionine uptake are effective in overcoming cisplatin resistance in an immunodeficient model, yet they fail to do so in an immunocompetent model. It shows that there is an undiscovered mechanism between circRNA and cancer that affects the role of circRNA in cancer. Notably, the therapeutic potential of circ-CDYL-targeted therapies in combination with ICIs for improving patient outcomes in hepatocellular carcinoma (HCC) is clinically significant. Targeting circCCNY has important clinical value for overcoming lenvatinib resistance in patients with hepatocellular carcinoma (HCC). CircNDUFB2, functioning as a potent RIG-I agonist, may hold promising potential for the immunotherapy of NSCLC.

Although the mechanism by which circRNAs regulate protein ubiquitination remains to be elucidated, nevertheless, it is anticipated that investigations into circRNA-based therapeutic strategies will lay a foundational groundwork for novel approaches to drive the advancement of precision cancer treatment in the future.

## 8 Conlusion

On this basis, the modulation of protein ubiquitination by circRNAs is likely a pivotal molecular mechanism underlying cancer pathogenesis and progression. On one hand, circRNAs can promote the ubiquitination and subsequent degradation of target proteins, directly contributing to the breakdown of these proteins and influencing cancer initiation and progression. On the other hand, circRNAs can inhibit the ubiquitination and degradation of target proteins, thus impeding the degradation of these proteins and affecting cancer development. A more in-depth understanding of the interaction mechanisms between ubiquitination and circRNAs, as well as the specific roles of circRNAs in cancer, may enable the discovery of more effective cancer diagnostic and therapeutic strategies, ultimately improving patients’ quality of life and survival rates.
